# Evaluation of Smartphone Inertial Sensor Performance for Cross-Platform Mobile Applications

**DOI:** 10.3390/s16040477

**Published:** 2016-04-04

**Authors:** Anton Kos, Sašo Tomažič, Anton Umek

**Affiliations:** Faculty of Electrical Engineering, University of Ljubljana, Ljubljana 1000, Slovenia; saso.tomazic@fe.uni-lj.si (S.T.); anton.umek@fe.uni-lj.si (A.U.)

**Keywords:** smartphone sensors, mobile application, participatory sensing, Internet of Things, cross-platform application, inertial sensor performance parameters

## Abstract

Smartphone sensors are being increasingly used in mobile applications. The performance of sensors varies considerably among different smartphone models and the development of a cross-platform mobile application might be a very complex and demanding task. A publicly accessible resource containing real-life-situation smartphone sensor parameters could be of great help for cross-platform developers. To address this issue we have designed and implemented a pilot participatory sensing application for measuring, gathering, and analyzing smartphone sensor parameters. We start with smartphone accelerometer and gyroscope bias and noise parameters. The application database presently includes sensor parameters of more than 60 different smartphone models of different platforms. It is a modest, but important start, offering information on several statistical parameters of the measured smartphone sensors and insights into their performance. The next step, a large-scale cloud-based version of the application, is already planned. The large database of smartphone sensor parameters may prove particularly useful for cross-platform developers. It may also be interesting for individual participants who would be able to check-up and compare their smartphone sensors against a large number of similar or identical models.

## 1. Introduction

Mobile applications are a very important segment in the software market and the competition in the mobile application market is fiercer every year. The need for rapid application development and deployment has never been greater. The development of mobile applications is becoming more challenging with the availability of many different platforms and their dedicated single-platform software development kits. In the single-platform development concept, an application is first developed for a specific platform, and if successful, it may later be developed for other platforms. This concept is giving way to the cross-platform application development, where an application is simultaneously developed for several platforms. To reduce the cross-platform development costs and to reach to as many users as possible, the development is shifting to cross-platform development tools. In regard to the abovementioned, such tools will become more and more important in the coming years.

In recent years the majority of mobile applications have been developed primarily for smartphones. Today’s smartphones incorporate numerous sensors. Depending on the particular smartphone model, the sensor count may include an accelerometer, gyroscope, compass, GPS, microphone, camera, proximity sensor, light sensor, temperature sensor, pressure sensor, *etc*. The abundance of different sensor types and sensor count heterogeneity of smartphone models are posing a great challenge to the developers of cross-platform applications. One of the challenges is also the variability of parameters of a particular sensor type in various smartphone models. A cross-platform application should be able to work with any targeted sensor type used in any targeted smartphone platform. Therefore, a developer should be well aware of the performance parameters of a particular type of sensors that can potentially be used in the application being developed for targeted smartphone platforms. Smartphone applications cannot directly access physical sensors embedded into smartphones. Raw sensor signals from physical sensors are processed by the smartphone’s operating system (OS) and made available to applications in a standardized format as a *smartphone sensor*. In this work, the focus is on accelerometer and gyroscope sensors, which are micro-electro-mechanical system (MEMS) sensors. In the remainder of this paper the term *sensor* denotes the smartphone sensor defined above, and the term *MEMS sensor* denotes the smartphone-embedded physical sensor. Developers should be aware that the applications can only access smartphone sensor data and not sensor data from the MEMS sensor chip directly, therefore sensor parameters cannot be taken from the MEMS sensor manufacturer’s data sheet.

Smartphone sensors are being employed in a growing number of mobile applications. Among these, mobile sensing applications are playing an increasingly important role in many aspects of our lives, such as health, fitness, sports, social networking, environmental monitoring, public infrastructure, navigation, and urban sensing [1,2,3,4,5,6]. The concept of mobile sensing has been identified as an appropriate technique for our objective—the evaluation of smartphone inertial sensors performance for cross-platform mobile applications.

Mobile sensing applications are designed and used at different scales [7]. *Personal sensing applications* are aimed and designed for an individual, with typical applications in wellbeing and recreation. Generated data are usually not shared with others. *Group sensing applications* are designed for a limited number of participants cooperating to achieve a common goal, for example, monitoring the garbage in the neighbourhood [7]. *Community sensing applications* include a large number of participating people. They offer collection, analysis and sharing of large amounts of data. An important issue with sensing applications is to what extent they require an active participant involvement. Two sensing paradigms are identified [7]: (a) *participatory sensing*, where a participant is actively engaged in the data collection and (b) *opportunistic sensing*, where the data collection is done automatically by the sensing application.

Mobile sensing applications require differing degrees of sensor data quality. While some do not require highly accurate sensor data, others depend on it. For example, an application for daily activity monitoring, using accelerometer data, primarily needs the information when a person is moving and in what patterns, hence accurate sensor data values are less important. A contrary example is the wheelchair accessibility application that uses smartphone inertial sensor data to track movements and path [8]. For accurate tracking a precise and accurate sensor readings are required during the the entire application activity. Similarly, accurate accelerometer readings are needed for measuring structural vibrations [9]. Other similar applications of both kinds can be identified in [1,2,3,4,5,6,7,8,9,10].

Mobile sensing applications fit perfectly into the Internet of Things (IoT) and Internet of Everything (IoE) paradigms. While the sensing capability of IoT devices is generally limited to only one or a few quantities, smartphones are less limited in this respect. Because smartphones include plenty of different sensors, have good communication capabilities, and are closely linked to their owners and owners’ activities, they play an increasingly important role in the IoT and IoE.

Mobile applications on smartphones are already used in many spheres of our daily life. Some important areas of use, that are expected to grow rapidly in the near future, are eHealth and eCare. Algorithms that are able to detect the various states of patients or persons under care are an important field of research [11]. In some cases one’s life can even depend on the correct and timely detection of his or her state. Application developers must take special care to develop detection algorithms that are robust enough to prevent most of the possible adverse events. Smartphone sensor parameters in changing environmental conditions may differ greatly from parameters measured in a controlled environment. It is therefore very important to measure smartphone sensors in daily use under changing environmental conditions. Such measurements better define the upper bounds of sensor parameter deviations and offer developers better information about safety factors needed in their applications. We are convinced that the information about smartphone sensor parameters can be very helpful for research and application development community, as the researchers and developers have to be aware of the reliability of the data they are using, especially when developing eHealth applications, where whether we like it or not, smartphone sensors are and will be used.

New smartphone models are being introduced at a rapid pace and participatory sensing seems to be the only feasible way of quantifying their performance in real-use conditions. Participatory sensing measurements are performed in uncontrolled environments and they yield sensor parameter deviation boundaries in real-use conditions. Such real-use parameters are the information about smartphone sensor quality that is of great assistance to cross-platform mobile application developers.

The paper is structured in the following way: Section 2 presents the motivation for this work and lists the most important contributions; related work is also discussed. Section 3 describes the implementation of the pilot participatory sensing system and its architecture. Measurement protocols and methodology are also defined. The results gained through the pilot application are presented in Section 4. Discussion and future work are covered in Section 5. We conclude in Section 6.

## 2. Motivation, Contributions and Related Work

Our research group has been studying and developing biomechanical biofeedback systems and applications based on inertial sensors. We have learned that various biofeedback applications have various demands concerning sensor data accuracy and precision. These demands are expressed through boundary values of parameter errors induced by sensor inaccuracy and imprecision. Two of the several factors that contribute to the sensor errors are bias and noise. These error sources induce parameter value errors that have different dependency on the duration of the analysis. For example, the angular error induced by gyroscope bias has a linear dependency on time and the position error induced by the accelerometer has a quadratic dependency on time. To estimate if a specific sensor is good enough for a specific application, the quality of the smartphone sensor must be known.

Our primary motivation, based on experience from biofeedback applications, is to design and implement a mobile sensing application that allows measurement, analysis, and storage of smartphone sensor parameters. Participatory sensing enables acquisition of large number of measurements in short time, therefore it is our method of choice.

The expected results and benefits of mobile participatory sensing application are numerous. They offer not only answers to purely research and academic questions but also solutions applicable to real-world problems and issues connected to the use of smartphone sensors in the development of a cross-platform applications. Cross-platform application developers can benefit from the statistical properties of measured sensors on which one can draw general conclusions about sensor’s quality and usability for various purposes and under various conditions and demands. One example of a practical benefit is the comparison of a particular measured sensor to other measured sensors of the same type already in the database. Such comparisons could identify damaged or deteriorated sensors or simply point to the fact that the measured sensor may be operating outside the normal operation conditions; for instance at the temperature that is outside of the recommended range. When the discussion is limited to smartphone sensors, several practical benefits can be identified. Some of them are listed below:

- Comparison of smartphones of the same platform.
- Comparison of smartphones of the same manufacturer.
- Comparison of the same smartphone model using different applications.
- Comparison of smartphone models with the same physical sensor, but on different platform.
- Comparison of results from the same physical smartphone through some period of time.

A possible obstacle for a successful implementation of the mobile sensing application is the recruitment of a large enough number of participants. Customary mobile sensing applications start as small-scale projects that are later expanded into full-scale applications. The small-scale phase is usually covered by recruiting a group of a few tens to a few hundreds of participants; in academic circles most commonly students. For a large-scale phase incentive mechanisms are needed [12]. For our mobile sensing application we identified a few incentive mechanisms for which we believe that could work: (a) participation in a research program; (b) immediate sensor quality information sent to the participant; absolute parameter values and comparison to other participants; and (c) gamification. Another obstacle is connected to measurements that are not performed in controlled conditions or measurements that deviate from the measurement protocol. Deviations from the expected results can be detected only to certain extent.

The contributions of this paper are:

- Confirmation of the usefulness of the participatory data acquisition concept for mass measurement and collection of smartphone sensor parameters. This is attained through the development, implementation, and employment of a pilot participatory application.
- Compilation of a database containing measured sensor parameters of more than 60 smartphone models. This is achieved through the recruitment of more than one hundred participants that performed more than 500 measurements of their smartphone sensors by using the pilot participatory sensing application.
- Useful results and findings about smartphone sensor properties and their statistical parameters that can be the base for directions to developers of cross-platform applications. One important result is the possibility of the identification of faulty devices that are, for example, potentially life-threatening if used in eHealth or eCare applications.

### Related Work

Participatory sensing applications are primarily designed for sensing physical quantities of interest, such as air pollution, temperature, body activity, and others [1,6,7,13]. In these applications smartphone sensors are used to measure the values of the quantity of interest. The primary focus is on gathering the sensor data, less attention is given to the quality of the acquired sensor data.

Participatory sensing applications with high data quality demands cannot use raw smartphone sensor data. They may include intolerable measurement errors that must be eliminated or reduced before the sensor data is used by the sensing application. Smartphone participatory sensing application for monitoring air pollution [13] requires high data quality. It calibrates the smartphone sensors by comparing their readings to governmental measurement stations when in their vicinity. Authors in [14] use iterative approach for calibration of smartphone sensors in monitoring pollution sources. In contrast to cooperative methods with neighbouring sensors and ground truth, their solution includes implicit calibration process in uncooperative environment.

In addition to sensor data quality, cross-platform developers should also consider the high variability of sensor parameters. The issues connected to variability of sensor parameters in smartphones are well described in [15]. The study is based on measurement of sensor parameters of nine different smartphone models. A number of papers focus on smartphone sensor performance evaluation and calibration. The authors of [16] performed laboratory measurements of accelerometer and gyroscope performance for the three high-end smartphones. The autonomous calibration of smartphone sensors based on sensor fusion is the main topic of paper [17]. Two different smartphone models are taken into consideration. Time and computation efficient calibration of accelerometers and gyroscopes is described also in [18].

To the best of our knowledge, no study that would use participatory sensing concept for evaluation of smartphone sensor quality has yet been conducted. The results of our study are available to anyone interested, for instance cross-platform application developers.

## 3. Pilot Participatory Sensing System

**Figure 1:**
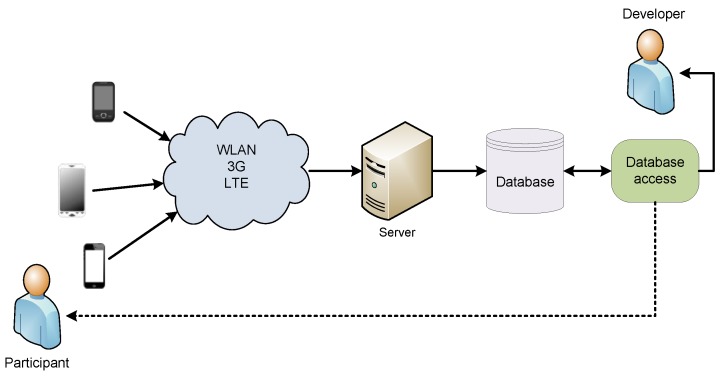
Pilot system architecture. Smartphones send sensor data over one of the available wireless interfaces to the processing computer (server). Processing results are stored in the database. They can be analysed and retrieved through a database access application.

The implemented pilot system employs the participatory sensing concept, where participants are actively engaged in the data collection. The architecture of the pilot system implementation is shown in Figure 1. The participants connect their smartphones to one of the available wireless networks and send sensor data to the server in the internet. The server extracts the measured sensor parameters and writes the anonymized results into the database. Participants can use the database access to check their personal results or various statistics. The pilot system is designed for a medium group of advanced smartphone participants.

In the pilot implementation a participant needs to install and setup one of the supported off the shelf applications that have the option to stream sensor data to a remote location. We used three different applications: (a) for the Android platform the *Sensor Node* version *1.53* (mScino Tools, Singapore, 2014) is used; (b) for iOS platform *Sensor stream version 1.1* (FNI Co., LTD, Seongnam, South Korea, 2013) and *Sensor monitor (Pro) version 1.0.9* (Young-woo Ko, Fuzz-Tech, Seoul, South Korea, 2010). Each subsequent sensor measuring episode must be actively started by the participant. By starting the measuring episode sensor data is sent to the server and anonymized results are written to the database. The server is in the public IP network and it is running the custom-designed LabVIEW™ application. The database used is XAMPP mySQL running on Windows server 2008 R2. For the retrieval of the participant’s smartphone measuring results, PhoneID is required. The pilot implementation has so far been used by our research colleagues and engineering students. They have all been educated about the correct measurement protocol.

### 3.1. Measurement Protocol

We focus on the measurement of a limited number of smartphone accelerometer and gyroscope parameters. Because the measurements are performed through the participatory sensing concept, they are performed by different subjects at different locations. The measurement protocol for the pilot participatory sensing application should be simple and straightforward. For example, it should not require the use of any hard-to-get tools or devices, it could be performed in less strictly controlled environment, each measurement episode should not take too long, *etc*. The above defined limitations impose a compromise between the complexity of the measurement protocol and the quality of measurements. We are aware of the fact that a looser measurement protocol produces lower quality results. Therefore we have chosen only a set of smartphone sensor parameters that can be measured with high enough reliability under the given protocol limitations. For smartphone accelerometer and gyroscope we measure their bias and a limited number of noise parameters.

The measurement of the sensor bias and noise parameters must be performed with the smartphone in a stand-still position. In addition to the aforementioned, the accelerometer bias measurement requires a levelled flat surface. All participants were instructed to use spirit level to ensure the measuring surface is appropriately levelled. The accuracy of standard commercial spirit levels is between 0.5 mm/m and 1 mm/m. Possible measurement error due to gravity projections on the axes parallel to the measuring surface are therefore up to 1 mg_0_. The smartphone must have the connection to the server. In each measurement episode the participant initiates sensor data streaming and puts the smartphone face-down onto the flat surface. Since the great majority of smartphones have flat screens, this position assures the standardized orientation of the smartphone. Assuming that the MEMS sensor axes are aligned with the smartphone screen and that the *Z*-axis is perpendicular to the screen, the gravity is expressed only in the *Z*-axis. It means that the gravity does not affect the readings of the *X* and *Y* axis of the accelerometer. The measurement begins after a few seconds of the detected stand-still position of the measured smartphone. During the measurement the participant must assure that the smartphone does not experience vibrations or movements of any kind. The measurement takes 100 s.

### 3.2. Measurement Methodology

The quality of smartphone sensors is limited by sensor inaccuracy and imprecision [19,20]. Sensor bias and noise cause parameter value errors that induce the linear angular error of gyroscope and quadratic position error of the accelerometer.

*Sensor bias* is defined as an average sensor output at zero sensor input. Bias value Equation (1) is estimated by averaging *N* samples of sensor signal. The bias estimate averaging time depends on sampling frequency *f_s_* and signal sample block length *N*. Bias estimate exhibits variations which are the result of a sensor noise:

(1) $$x_{bias}=\frac{1}{N}\sum_{n=0}^{N-1}x\left[n\right]$$

*Sensor noise* characteristics can be observed by measuring the *Allan variance*. Allan variance Equation (3) is defined as the average squared difference between successive bias estimates *y*[*m*] Equation (2). Each *y*[*m*] is calculated from a block of *N* signal samples [21]:

(2) $$y\left[m\right]=\frac{1}{N}\sum_{n=0}^{N-1}x\left[n+m\cdot N\right]$$

(3) $$\sigma_{A_{}}^{2}\left[N\right]=\frac{1}{2}\bar{{\left(y\left[m\right]-y\left[m-1\right]\right)}^{2}}$$

Allan variance is estimated from a finite data stream in *M* successive bias estimates *y*[*m*] Equation (3):

(4) $$\sigma_{A_{}}^{2}\left[N\right]\approx \frac{1}{2\cdot (M-1)}\sum_{m=1}^{M-1}{\left(y\left[m\right]-y\left[m-1\right]\right)}^{2}$$

The approximation error of Equation (4) is [21]:

(5) $$\delta_{\sigma }=\frac{1}{\sqrt{2(M-1)}}$$

By definition Equation (3) the Allan variance is a function of block length *N*, which can also be expressed by the averaging time *T*_avg_ = *N*/*f_s_*. Sensor noise generally originates from different random processes. Various noise terms with different spectral power densities participate at the same time [21]. For averaging times around 1 s the white noise is most often a dominant error source for MEMS gyroscopes and accelerometers. In such cases the Allan variance measured at averaging time *T*_avg_ = 1 s represents the sensor white noise power density. Allan deviation σ_A_ at *T*_avg_ = 1 s is also known as *velocity random-walk* parameter (VRW) for accelerometer and *angular random-walk* parameter (ARW) for gyroscope [22,23].

Pilot participatory application calculates sensor biases Equation (1) by averaging *N* = 300 sensor samples at sampling frequency *f_s_* = 30 Hz. The corresponding bias estimate averaging time is therefore 10 s. The sampling frequency is chosen as the greatest common sampling frequency accepted by all smartphone applications listed at the beginning of Section 3. Calculated bias estimates of each measurement are stored in the database. Accelerometer biases are given relative to the gravity constant *g*_0_. Gyroscope biases are given in rad/s.

Allan variance function is commonly presented in a graph. Its calculation requires a large number of signal samples and consequently long measurement times. For the statistically relevant results (Equation (5)) the measurement time must be at least ten times longer than the largest averaging time *T*_avg_ presented in the function graph. For example, if the maximal averaging time presented in the graph is 100 s and *M* = 10 the measurement takes more than 16 min. One of the limitations of participatory sensing protocol from Section 3.1 is that the measurement should not take too long. Instead of measuring the Allan variance function, we chose to measure only the characteristic point at *T*_avg_ = 1 s that represents sensor white noise power density parameter. The validity of presumed white noise model around *T*_avg_ = 1 s was confirmed by a number of preliminary measurements of various smartphone models.

Pilot participatory application calculates Allan variation σ_A_ (Equation (4)) from *M* = 100 clusters with *N* = 30 samples at sampling frequency *f_s_* = 30 Hz. Noise parameter measurement takes 100 s. The predicted relative measurement error δ_σ_ = 0.071 (Equation (5)). Calculated noise parameters of each measurement are stored in the database. Accelerometer noise parameters (VRW) are given in *g*_0_/$\sqrt{\text{Hz}}$. Gyroscope noise parameters (ARW) are given in deg/s/$\sqrt{\text{Hz}}$.

## 4. Results

In the implemented pilot participatory sensing application we have managed to collect over 500 measurements of 116 different smartphone devices in 44 days. The collection of smartphones includes 61 different models of which 31 models are measured once and 30 models more t. The smartphone models that were measured the most times were: Galaxy S3, Galaxy S4, iPhone 4, iPhone 5, iPhone 5S, iPhone 6, Nexus 5, and Xperia Z1 Compact. They are included in the statistics by model (see Section 4.2). The measured smartphone models come from 13 different manufacturers and use two different platforms. Measurement results are presented in three scales: (a) complete dataset results; (b) results by smartphone model; and (c) results of individual smartphone devices.

### 4.1. Complete Dataset Results

**Figure 2:**
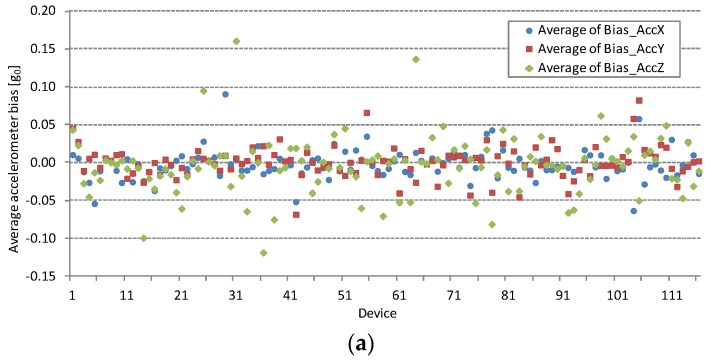

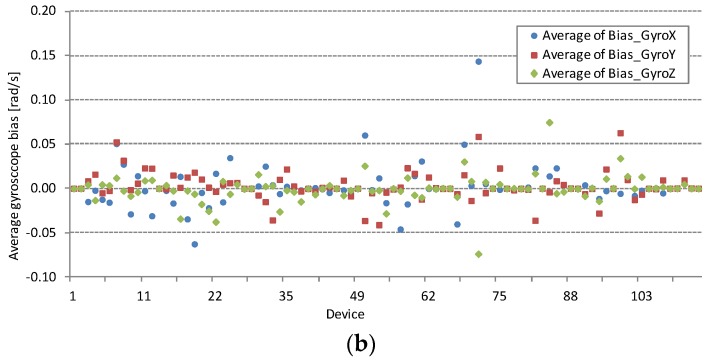
Accelerometer and gyroscope bias measurements of *X*, *Y* and *Z* axes. (**a**) Average accelerometer biases of 116 different smartphones are plotted; (**b**) Average gyroscope biases of smartphones with gyroscopes are plotted. The horizontal axis represents the device identification number (ID) from the measurement database.

**Figure 3:**
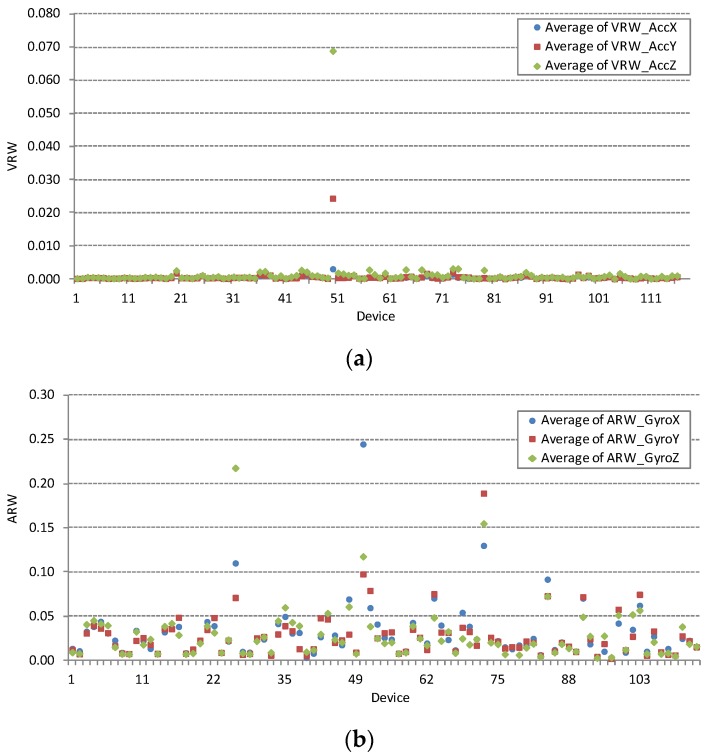
Accelerometer and gyroscope noise measurements of the axes *X*, *Y* and *Z*. The horizontal axis represents the device identification number from the measurement database. (**a**) The complete accelerometer measurement dataset is plotted, including an example of a faulty device with ID = 50 that evidently deviates from other devices. The vertical axis shows VRW in [g_0_/$\sqrt{\text{Hz}}$]; (**b**) The complete gyroscope measurement dataset is plotted, including the examples of the three faulty devices with ID = {25, 50, 73}. The vertical axis shows ARW in [deg/s/$\sqrt{\text{Hz}}$].

**Figure 4:**
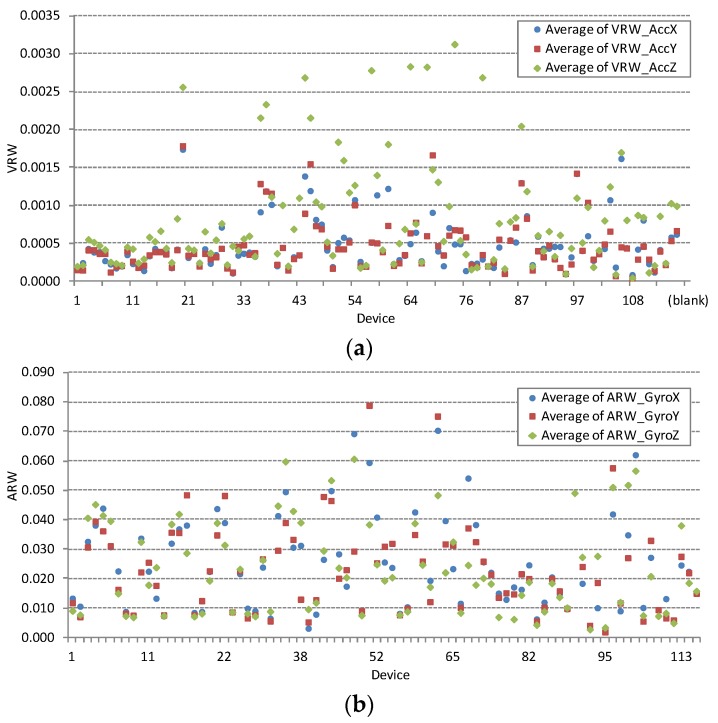
Accelerometer (**a**) and gyroscope (**b**) noise of all properly functioning devices (*K_a_* = 108, *K_g_* = 82). The horizontal axis represents the device identification number from the measurement database. Considerable deviations are the result of different models of MEMS sensor chips embedded in different smartphone models. The vertical axis shows (**a**) VRW in [g_0_/$\sqrt{\text{Hz}}$] and (**b**) ARW in [deg/s/$\sqrt{\text{Hz}}$].

In this subsection the complete measurement dataset is presented by the measured devices. Plotted values represent the averaged measured parameter values by the device. Figure 2 shows smartphone accelerometer and gyroscope biases, Figure 3 and Figure 4 their noise, and Table 1 their average, standard deviation, and percentiles.

Accelerometer and gyroscope biases are measured under conditions defined in the last paragraph of Section 3.2. The averaged accelerometer biases for all devices under test are shown in Figure 2a and averaged smartphone gyroscope bias measurements in Figure 2b. Some devices have large bias, but they are not necessary faulty. For example, excessive gyroscope bias values are evident in the results of the device with ID = 73 in Figure 2b.

Accelerometer and gyroscope biases should be measured and compensated in most sensor based applications. However, some less demanding applications can work even without bias compensation. The statistics on absolute bias values is collected in Table 1. Absolute bias values for more than 50% of measured devices stay under average and 95th percentile might be helpful for cross-platform application developers. More detailed results will be acquired after starting a planned full-scale cloud-based application. At least ten times larger testing group will provide results with higher statistic relevance for higher percentiles, for example 99th percentile.

Accelerometer noise parameter VRW and gyroscope noise parameter ARW are obtained from single point Allan variation measurements under conditions defined in Section 3.1 and Section 3.2. Large deviations in measured values can be used to diagnose faulty devices. Such examples are visible in the complete VRV and ARW measurement dataset shown in Figure 3. Smartphone device with ID = 50 in Figure 3a exhibits enormous accelerometer noise density parameter VRW. Similarly, Figure 3b show three devices with ID = {25, 50, 73} with excessive gyroscope noise density parameter ARW. All listed faulty devices are excluded from further analysis. Figure 4 shows large deviations in measured noise parameters for both sensors after exclusion of the three identified faulty devices. Large deviations are the result of different noise characteristics of MEMS sensor chips embedded in different smartphone models. Noise parameter is further analysed after clustering the complete data set by smartphone model in the next section.

### 4.2. Results by Smartphone Model

**Figure 5:**
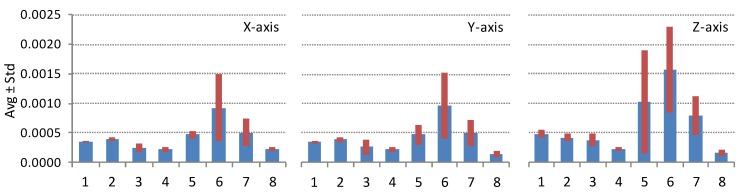
Comparison of average accelerometer noise parameter VRW and its standard deviation for eight different smartphone models. Smartphone models presented are: Galaxy S3, Galaxy S4, iPhone 4, iPhone 5, iPhone 5S, iPhone 6, Nexus 5, Xperia Z1 Compact. The listed smartphone models are presented in a random order (the same for all graphs). The vertical axis shows VRW in [g_0_/$\sqrt{\text{Hz}}$].

**Figure 6:**
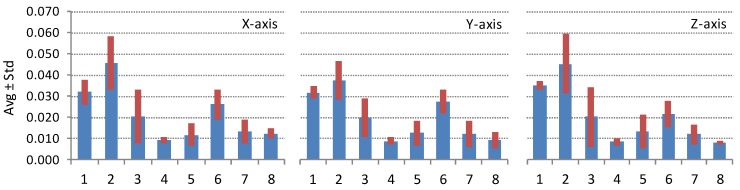
Comparison of average gyroscope noise parameter ARW and its standard deviation for eight different smartphone models. Smartphone models presented are the same as in Figure 5. The vertical axis shows ARW in [deg/s/$\sqrt{\text{Hz}}$].

While statistics of the complete measurement dataset gives us the overall picture of the smartphone sensor statistics, it is sometimes more interesting to filter out the results for a particular smartphone model. In this subsection the statistics of different smartphone models are presented and compared. Only the smartphones with enough different devices of the same model have been taken into consideration. The selection includes eight popular smartphone models: Galaxy S3, Galaxy S4, iPhone 4, iPhone 5, iPhone 5S, iPhone 6, Nexus 5, Xperia Z1 Compact. The same set of smartphone models is used in Figure 5 and Figure 6.

Noise parameter values, VRV and ARW, are strongly connected to the embedded MEMS chip technology. For illustration we group the dataset of properly working devices shown in Figure 4 by the smartphone model. The comparison is done for the popular smartphone models listed in the previous paragraph. At least six different devices of the same model are measured. Figure 5 shows the comparison of the averaged accelerometer noise density VRV in combination with its standard deviation and Figure 6 shows the comparison of the averaged gyroscope noise parameter ARV in combination with its standard deviation; the same set of smartphones is used in both figures.

Results in Figure 5 and Figure 6 are presented in the same random order. Similar differences in sensors noise parameters between various smartphone models are evident for all principal axes. Smartphone models with codes 4 and 8 exhibit the best performance of both sensors. While smartphone models with codes 1, 2 and 3 show better performance for accelerometer and below average performance for gyroscope, smartphone with code 7 shows the opposite, better performance for gyroscope and below average performance for accelerometer.

### 4.3. Results by Individual Device

**Figure 7:**
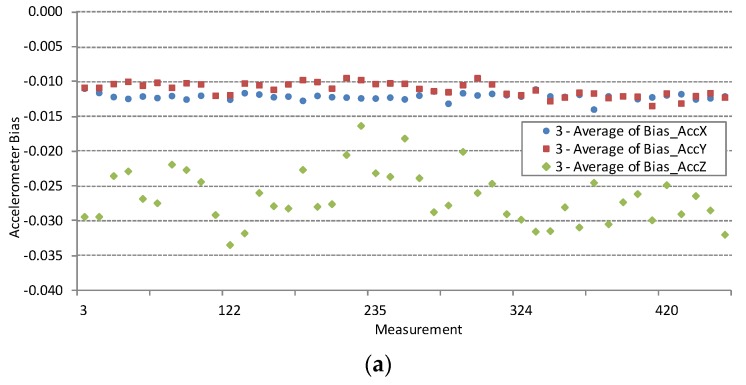

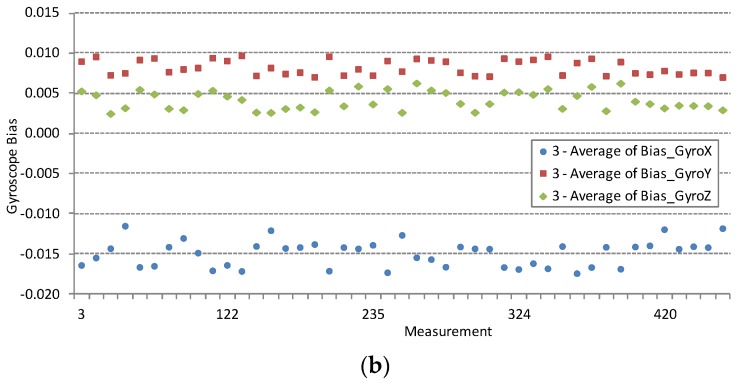
Repetitive accelerometer and gyroscope bias measurements (*N* = 44) of the smartphone with ID = 3 showing bias variation. Measurement numbers are taken from the database and are not successive as other measurements took place in between two measurements of the presented device. Accelerometer bias is in [g_0_], gyroscope bias is in [rad/s].

The results of the complete dataset and statistics by model, presented in Section 4.1 and Section 4.2, are particularly useful for cross-platform developers. Individual participants may find these results useful when comparing their smartphone device to all the smartphones measured or to the smartphones of the same model, brand, platform, *etc*. But participants, who measure their device repeatedly, over some period of time, can benefit also from the statistics about their own device. Figure 7 presents one such case when the participant is measuring his/her device repeatedly 44 times. Table 2 and Table 3 list the statistics for several smartphone devices that were measured more than 10 times over the period of 44 days.

A large number of bias measurements for the same sensor device can give some information about one of the most important sensor parameter; bias variation. Accelerometer and gyroscope biases vary with time. Bias variations are the result of random, low-frequency sensor noise and of deterministic dependence on temperature fluctuations. Deterministic bias drift cannot be compensated without measuring of sensor temperature.

Several smartphones have been repeatedly measured during the 44 days testing period. Figure 7a,b show the results of *N* = 44 simultaneous accelerometer and gyroscope bias measurements of the iPhone 4 smartphone device with ID = 3. This device was under the test every evening repeatedly for 44 days. Bias variations pattern show that considerable variation is observed on a daily scale.

To show bias variations on individual device a group of five devices of different smartphone models is selected: Xperia Z1 Compact (ID = 2), iPhone 4 (ID = 3), iPhone 5S (ID = 7), iPhone 6S (ID = 8), and LG Nexus 5 (ID = 14). Measurement statistics for the accelerometer biases of the above listed smartphone is presented in Table 2 and statistics for the gyroscope biases in Table 3. Bias variation is not critical for all devices under test. For example, device with ID = 14 from Table 2, exhibits large accelerometer bias, but very small bias variation. Devices ID = 2 and ID = 14 from Table 3 exhibit negligibly small gyroscope bias and bias variation.

## 5. Discussion and Future Work

The results of our pilot implementation are interesting and encouraging. Even in this limited volume, they can prove useful to developers of cross-platform sensing applications. Larger scale measurements would give us a better overall picture of existing smartphone sensor’s performance. Its results would form a basis for further research in this field, such as data mining, and offer a good reference for the development of cross-platform smartphone sensing applications.

The obvious next step is a planned upgrade to a full-scale cloud based participatory sensing application. The application would operate in the cloud with open public access to anonymized and statistically processed results and personal access to user’s own data. Deployment of such participatory sensing application is a complex task. Concepts such as Sensing as a Service (S^2^aaS) [12] have been proposed to ease the task of designing mobile sensing applications. Platforms, such as described in [24,25,26], can further help developers to avoid inventing the wheel and enable them to implement their applications in a shorter period of time.

**Figure 8:**
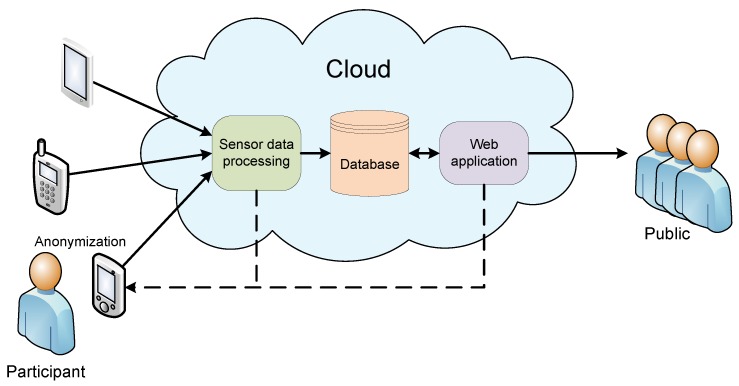
Cloud based participatory sensing system architecture. Smartphones send sensor data to the cloud for processing. Processed sensor data results are stored in the cloud database. Results can be retrieved directly from the processing unit or through a cloud web application.

The general architecture of the planned next-step participatory sensing system is shown in Figure 8. It is similar to the architecture of many other cloud applications. The system employs the participatory sensing paradigm, where participants are actively engaged in the data collection. Before the first use, a participant must install the custom developed sensing client onto the smartphone. Also, each subsequent sensor measuring episode must be actively started by the participant. By starting the measuring episode, sensor data is sent to the processing instance in the cloud. Processing results are then written to the cloud database. A cloud web application is used for data retrieval from the database and its statistical analysis. The public has access to the anonymized results in the database. Participants can retrieve their results from the data processing unit immediately after the measuring episode, or later from the cloud web application.

On a longer run, the presented system could be upgraded with functionalities other than measurement and statistical analysis of sensor performance. For example, less demanding biofeedback applications could use the existing infrastructure for sending sensor data to the biofeedback processing unit, which would process the data and send the biofeedback signals back to the participant. Still further, the system could be supplemented by the opportunistic functionality; taking measurements periodically, with prior consent of the participant, at times favourable for measurements (during the night, longer standstill, *etc*.). Not all sensor parameters can be measured through the opportunistic paradigm as some measurements require the participant’s actions during the measurement

By collecting large amounts of data from a large number of participants, privacy is of the prime concern [26] and participatory sensing application must provide sufficient measures to prevent any concerns about the issue. The presented sensing system will perform anonymization inside the installed smartphone application; hence all the data leaving the phone will be anonymized. The smartphone identity, in the form of a PhoneID hash, will be known only to the participant initiating the measuring episode. The same hash is needed when the participant wants to get the results from the cloud web application. The public sees only the anonymized and statistically processed results.

## 6. Conclusions

Do you know how good your smartphone’s sensors are? Having in mind that smartphone sensor parameters do not directly reflect the embedded physical sensor’s parameters, measuring the former is an important task. The results gathered by the pilot participatory sensing application reveal that there is no straightforward answer to the above question and that the parameters of measured smartphone sensors vary considerably between different smartphone models and some of the parameters also within the same model. By closer inspection of individual measured sensor parameters it can be seen that while biases vary between the smartphone models and within the same model, noise varies between the smartphone models and is stable within the same model.

How can a developer of a cross-platform application benefit from such results? Knowing that different applications require different levels of sensor quality, that bias can be compensated, and that noise can only be reduced, but not eliminated, a developer may choose different approaches. For example, if the application requirements are low, the developer may decide not to compensate biases at all, providing that the targeted group of smartphones exhibit biases that satisfy application requirements.

The results of the pilot application give us the first insight of the statistics of smartphone accelerometer and gyroscope bias and noise. Extending the number of participants, smartphone models, manufacturers, sensor parameters, and platforms, would improve the statistical relevance of the results. Plenty of other options and ideas for upgrades and improvements to the pilot application are already in the scope. The first in the list is the upgrade to a full-scale cloud-based application. It will offer the inclusion of more measurements, other smartphone sensors and their parameters, that would further improve the usability of already gained results.

## Figures and Tables

**Table 1:** Statistical parameters of the measured smartphone accelerometer and gyroscope biases. Average, standard deviation and several percentiles for absolute bias values are listed.

	Accelerometer [mg_0_]			Gyroscope [mrad/s]		
Parameter	*X*	*Y*	*Z*	*X*	*Y*	*Z*
Average	14.3	14.6	25.3	9.4	8.7	6.1
StDev	14.2	15.2	25.1	13.6	12.1	8.7
50th percentile	10.0	9.9	18.5	3.1	4.3	2.8
90th percentile	30.1	31.5	60.3	30.8	22.9	17.1
95th percentile	43.6	45.9	71.1	40.5	35.4	28.2
100th percentile	90.9	82.7	161.0	142.7	81.7	158.2

**Table 2:** Statistical parameters of smartphone accelerometer bias. Selected devices with more than 10 measurements are presented. Average and standard deviation [g_0_] for the three axes are listed.

		Average			Standard Deviation			Max–Min		
ID	*N*	*X*	*Y*	*Z*	*X*	*Y*	*Z*	*X*	*Y*	*Z*
2	41	0.0063	0.0279	0.0231	0.0057	0.0019	0.0018	0.0236	0.0065	0.0075
3	44	−0.0121	−0.0110	−0.0264	0.0005	0.0010	0.0038	0.0030	0.0040	0.0171
7	42	0.0051	0.0060	0.0034	0.0009	0.0012	0.0011	0.0045	0.0046	0.0056
8	14	0.0029	0.0032	0.0004	0.0025	0.0009	0.0008	0.0097	0.0025	0.0030
14	11	−0.0241	−0.0259	−0.0994	0.0006	0.0017	0.0013	0.0017	0.0058	0.0046

**Table 3:** Statistical parameters of smartphone gyroscopes bias. Selected devices with more than 10 measurements are presented. Average and standard deviation [rad/s] for the three axes are listed.

		Average			Standard Deviation			Max–Min		
ID	*N*	*X*	*Y*	*Z*	*X*	*Y*	*Z*	*X*	*Y*	*Z*
2	41	0.00000	0.00002	0.00001	0.00013	0.00009	0.00010	0.00051	0.00042	0.00045
3	44	−0.01495	0.00833	0.00423	0.00158	0.00091	0.00116	0.00591	0.00268	0.00380
7	42	0.05062	0.05244	0.01190	0.00396	0.00054	0.00133	0.01656	0.00221	0.00812
8	14	0.02728	0.03175	−0.00258	0.00054	0.00134	0.00037	0.00161	0.00410	0.00130
14	11	−0.00004	0.00002	0.00001	0.00016	0.00036	0.00013	0.00052	0.00142	0.00039
